# Effects of salidroside on atherosclerosis: potential contribution of gut microbiota

**DOI:** 10.3389/fphar.2024.1400981

**Published:** 2024-07-17

**Authors:** Si-Fan Fei, Can Hou, Fang Jia

**Affiliations:** Department of Cardiovascular Medicine, The First People’s Hospital of Changzhou, The Third Affiliated Hospital of Soochow University, Changzhou, China

**Keywords:** salidroside, atherosclerosis, gut microbiota, trimethylamine noxide, lipopolysaccharide, short-chain fatty acids

## Abstract

Much research describes gut microbiota in atherosclerotic cardiovascular diseases (ASCVD) for that the composition of the intestinal microbiome or its metabolites can directly participate in the development of endothelial dysfunction, atherosclerosis and its adverse complications. Salidroside, a natural phenylpropane glycoside, exhibits promising biological activity against the progression of ASCVD. Recent studies suggested that the gut microbiota played a crucial role in mediating the diverse beneficial effects of salidroside on health. Here, we describe the protective effects of salidroside against the progression of atherosclerosis. Salidroside regulates the abundance of gut microbiotas and gut microbe-dependent metabolites. Moreover, salidroside improves intestinal barrier function and maintains intestinal epithelial barrier function integrity. In addition, salidroside attenuates the inflammatory responses exacerbated by gut microbiota disturbance. This review delves into how salidroside functions to ameliorate atherosclerosis by focusing on its interaction with gut microbiota, uncovering the potential roles of gut microbiota in the diverse biological impacts of salidroside.

## Graphical Abstract

**Figure:**
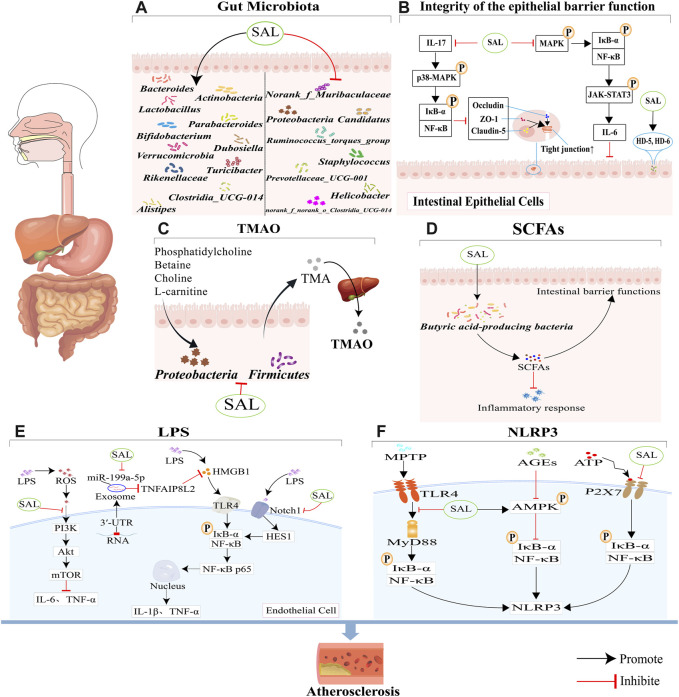
This review seeks to offer a comprehensive look at how salidroside impacts gut microbiota and its potential therapeutic role in treating atherosclerosis. **(A)** Salidroside has been shown to have a positive impact on atherosclerosis by promoting the growth of beneficial bacteria and decreasing the levels of harmful bacteria in the body. **(B)** Salidroside has been shown to enhance the integrity and function of the intestinal mucosal barrier through its ability to suppress NF-κB and p38 MAPK signaling pathways, modulate the NF-κB/MAPK/JAK-STAT3 signaling pathways and increase the expression of antimicrobial peptides HD-5 and HD-6. **(C)** Salidroside can reduce TMAO production through reducing the abundance of *Firmicutes* and *Proteobacteria*. **(D)** Salidroside can improve the expression of SCFAs, through increasing the abundance of some special bacteria. **(E)** Salidroside can reduce LPS-induced inflammation, which is associated with the inhibition of the ROS-mediated PI3K/AKT/mTOR signaling pathway, the downregulation of exosome miR-199a-5p, and the attenuation of the Notch-Hes signaling pathway. **(F)** Salidroside can inhibit NLRP3-associated gut-coronary axis, including TLR4/MyD88/NF-κB/NLRP3 signaling pathway, AMPK/NF-κB/NLRP3 signaling pathway, and P2X7/NF-κB/NLRP3 signaling pathway.

## 1 Introduction

Atherosclerosis is the underlying disease process for the emergence and progression of atherosclerotic cardiovascular diseases (ASCVD). Despite significant advancements in preventing and treating cardiovascular disease, the mortality rate from ASCVD continues to rise in China, with stroke and ischemic heart disease being the primary causes of death in the country (Zhou et al., 2019). ASCVD is responsible for approximately two-thirds of arteriosclerosis-related deaths worldwide (Stone et al., 2022). Findings from the 2019 global burden of disease (GBD) study, which analyzed data from 204 countries spanning from 1990 to 2019, revealed a significant rise in the prevalence and mortality rate of cardiovascular disease (Roth et al., 2020).

Recent studies show that the composition of gut microbiota plays a crucial role in affecting cardiovascular health (Verhaar et al., 2020). Disruptions in the balance of the intestinal microbiome have been linked to various metabolic disorders such as diabetes, obesity, dyslipidemia, and depression. In addition, these imbalances may also contribute to the development of ASCVD and its related complications (Witkowski et al., 2020; Fan and Pedersen, 2021).

Genome sequencing of fecal microbiota reveals that variations in the gut metagenome may be linked to symptomatic atherosclerosis in individuals with unstable plaque. This finding suggests that the presence of specific microbial communities and metabolites in the gut may play a role in exacerbating inflammation, leading to atherosclerosis progression and potentially increasing the risk of cardiovascular events in certain individuals (Karlsson et al., 2012). The expression and regulation of intestinal microorganisms and their metabolites are influenced by various environmental factors and pathophysiological conditions. Therefore, gaining a deeper insight into the involvement of gut bacteria and its metabolites in the prolonged progression of atherosclerosis could offer innovative therapeutic tactics to enhance the outcomes of individuals with ASCVD.

Recent research has focused on investigating drug interventions to regulate gut microbiota and metabolites for treating ASCVD. Due to their fewer adverse effects, lower cost (Yang et al., 2023), and broad structural diversity and biodiversity (Cheng et al., 2022), natural compounds are considered a promising source of leading compounds with cardiovascular protective bioactivity. Therefore, natural compounds have shown promise in the treatment of ASCVD and metabolic diseases (Zhao et al., 2021).

China is recognized as the primary region for the growth of *Rhodiola*, boasting 73 species, two subspecies, and seven varieties. Approximately 90% of *Rhodiola* within China is concentrated in the northwest, southwest, and northeast regions (Zhuang et al., 2019). As early as 200 A.D., *Rhodiola* was documented as a medicinal botanical drug in both Tibetan and Chinese medical texts for treating cardiovascular diseases (Zhao et al., 2021). Salidroside is a phenol glycoside found in *Rhodiola* (Jin et al., 2022), which could be found in all *Rhodiola* species with concentrations ranging from 1.3 to 11.1 mg/g (Han et al., 2022). It can be prepared commercially, such as the Koenigs-Knorr method and enzymatic catalysis method (Zhang et al., 2021). Salidroside is known for its diverse range of benefits in the body, including boosting immunity, reducing inflammation, protecting against hypoxia, lowering blood sugar levels and preventing plateau reaction (Liu et al., 2023). However, its exact mechanism of action is still not completely understood. This review detailed how salidroside effectively addresses atherosclerosis by honing in on the gut microbiota. The potential impact of gut microbiota on the diverse health benefits associated with salidroside is also emphasized.

## 2 Anti-atherosclerotic effects

Atherosclerosis, a chronic inflammatory condition, is linked to dysfunction in endothelial cells, infiltration of lipids, recruitment of macrophages, and vascular smooth muscle cell migration (Zhang et al., 2021). Current studies have underscored the significance of gut microbiota along with its metabolites in the advancement of atherosclerosis, proposing fresh avenues for research and promising treatment strategies (Sanchez-Rodriguez et al., 2020; Vourakis et al., 2021). Salidroside, with significant biological activity demonstrated in various *in vitro* and in study (Zhang et al., 2021), emerges as a promising approach to decrease the risk of atherosclerosis-related diseases (Song et al., 2021). Salidroside is capable of improving the function of endothelial cells, inhibiting the proliferation of vascular smooth muscle cells, reducing lipid peroxidation, and preventing thrombus formation. It also has a positive impact on the integrity of the intestinal barrier and the composition of gut microbiota (Fei et al., 2023).

Numerous animal studies have demonstrated the potential of salidroside as a valuable therapeutic agent for ASCVD. Animal studies (Bai et al., 2019) demonstrated that salidroside could reduce atherosclerosis lesion formation, improve endothelial function and ameliorate inflammation. Six-week-old male ApoE^−/−^ mice were given a high-fat diet (HFD) for 8 weeks and treated with salidroside (25 and 50 mg/kg/d) at different doses for an additional 8 weeks (Xing et al., 2015). The atherosclerotic lesions, macrophage infiltration determined by CD68 immunostaining, MCP-1 and VCAM-1 expression were reduced in the salidroside (50 mg/kg/d) mice group compared with the HFD group. Salidroside, administered at a dose of 100 mg/kg/day, significantly reduced the development of atherosclerotic lesions and endothelial damage in mice with chronic intermittent hypoxia ApoE^−/−^ mice (Li et al., 2021). Salidroside has demonstrated its efficacy in enhancing lipid metabolism by lowering levels of fatty acids, cholesterol, and triacylglycerols in both the serum and liver of ApoE^−/−^ mice (Song et al., 2021). Salidroside has shown promise in slowing the advancement of atherosclerosis, but additional research is required to better understand its underlying molecular mechanisms, particularly in relation to the gut microbiota.

## 3 Bioavailability

Studying the various pharmacological effects of salidroside on atherosclerotic diseases through the perspective of intestinal microbiota is a hopeful area of investigation. The intestinal microbiome and liver are key sites for the metabolism of salidroside (Guo et al., 2014). Most salidroside is converted to glucuronic acid, sulfate derivatives, and aglycones and transported through the blood to target tissues (Luo et al., 2016). The bioavailability of salidroside is 32.1%–98% in rat plasma after intravenous and oral administration (Yu et al., 2008; Wen et al., 2020). The bioavailability of salidroside studied in an *in vitro* gastrointestinal digestion model was 98.7% and the bioavailability of salidroside studied in a Caco-2 cell model ranged from 2.10% to 2.68% (Zhou et al., 2018). The absolute bioavailability of salidroside varies from 32.1% to 98% in rodents. For instance, the absolute bioavailability value of Sal is evaluated as 98.0% at doses of 25 mg/kg (po.) and 5 mg/kg (i.v) administration, while 51.97% at doses of 100 mg/kg (ig.) and 50 mg/kg (i.v) administration (Fan et al., 2020). Salidroside undergoes a low level of biotransformation in the intestinal wall (Luo et al., 2016), but it is efficiently converted by the intestinal microbiome and liver (Luo et al., 2016).

**FIGURE 1:**
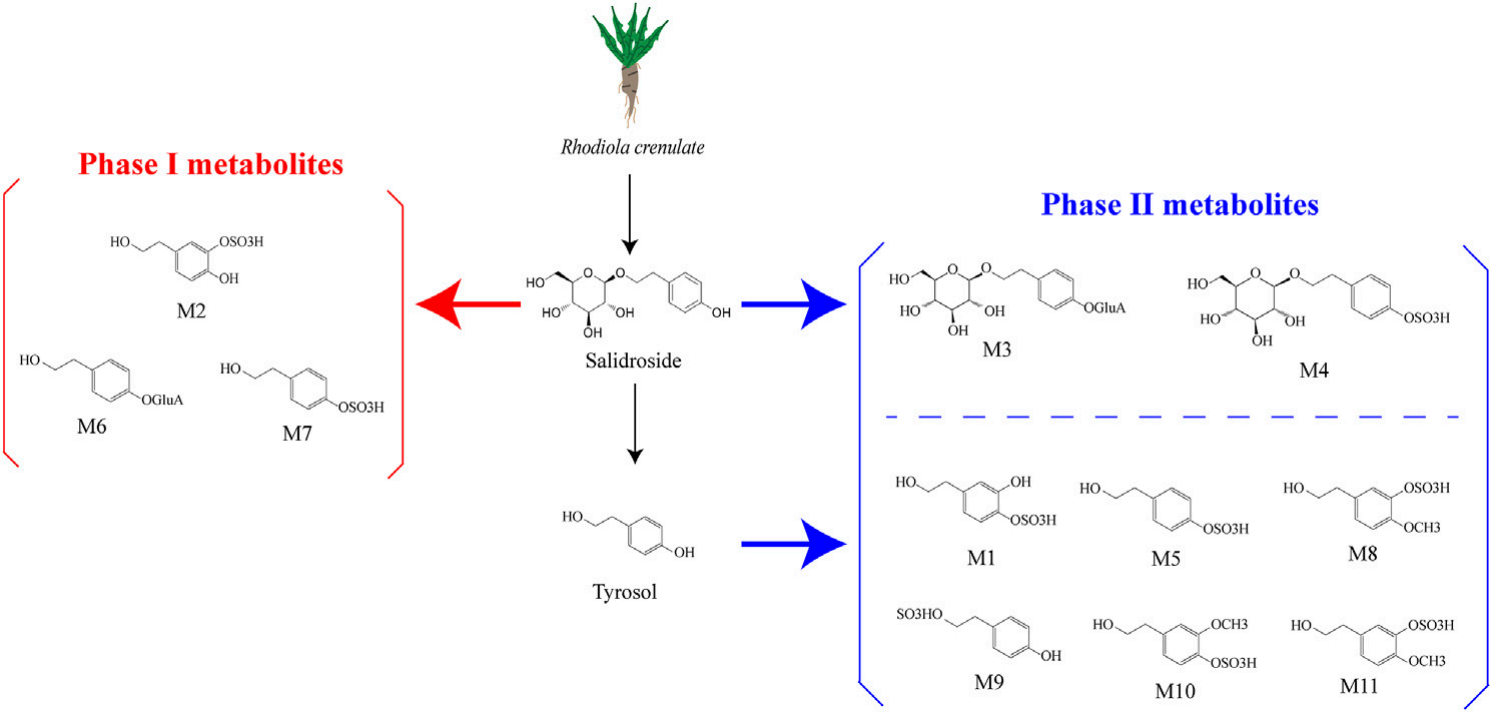
Salidroside and the metabolites (Han et al., 2016; Ke et al., 2020). Salidroside underwent both phase I and phase II metabolic reactions. The phase I biotransformation pathways included hydroxylation and dehydrogenation, resulting in metabolites M6, M7, and M2. On the other hand, phase II metabolism pathways involved sulfation and glucuronidation, leading to metabolites M3 and M4. Salidroside metabolizes to p-tyrosol, which likely undergoes hydroxylation, methylation, and dehydroxylation *in vivo* before being converted to phase II metabolites such as M1, M5, M8, M9, M10, and M11.

Research has shown that in rats following intragastric gavage, salidroside may undergo various metabolic reactions (Li et al., 2018). These reactions occur in two stages: Phase I and Phase II. Phase I metabolism involves various pathways such as hydrolysis, hydroxylation, deglycosylation, methylation, oxidation, and dehydrogenation. The phase I compounds M2, M6, and M7 of salidroside are biologically active, while another four phase II compounds of salidroside, M1, M3, M4, and M5, have no biological activity (Han et al., 2016). According to the results of a study, it was found that the main metabolic pathways of salidroside are glucuronidation, sulfation, and deglycosylation. Intestinal microbiomes can convert salidroside into p-Tyrosol (Luo et al., 2016). From this, we suppose that the I metabolic pathways of salidroside occur in the gut microbiota (Fan et al., 2020) (Figure 1).

## 4 Salidroside and gut microbiota

Atherosclerosis, a condition characterized by the buildup of plaque in the arteries, is closely linked to various risk factors such as hypertension, abnormal cholesterol levels, smoking, diabetes, and obesity. Recent study has been established that intestinal microbiome dysbiosis is an important factor in the development of atherosclerosis (Verhaar et al., 2020). Studies have shown that certain bacteria, such as *Collinsella*, *lactobacilli*, *Escherichia-Shigella*, and *Enterococcus*, are more prevalent in patients with coronary heart disease (CHD). On the other hand, beneficial bacteria like *Roseburia*, *Eubacterium* spp., *Bacteroides*, and butyrate-producing bacteria such as *Faecalibacterium*, *Roseburia*, and *Eubacterium rectalae* are found in lower levels in individuals with atherosclerosis. This suggests that the composition of gut microbiota plays a crucial role in the development and progression of atherosclerosis (Iatcu et al., 2021). *Veillonella*, *Haemophilus* and *Klebsiella* had a higher abundance in coronary artery disease (CAD) patients, which could induce endotoxemia and systemic inflammation (Liu et al., 2019).

In patients with acute myocardial infarction (AMI), the presence of *Parabacteroides merdae* was found to be more prominent. *A. muciniphila*, *E. hallii*, and *Roseburia hominis* were decreased in AMI (Liu et al., 2022). The levels of *Firmicutes* were lower in AMI patients compared to healthy controls, while *Bacteroidetes* showed a slight increase (Han et al., 2021). HFD-fed mice showed dramatic enrichment in *Desulfovibrio*, *Lachnospiraceae_NK4A136_group*, which were positively correlated with reactive oxygen species (ROS) (Wang et al., 2020). Additionally, the metabolic syndrome (MetS) mice model showed an increase in vascular inflammation and cardiovascular disease, which was linked to a lower ratio of *Firmicutes*/*Bacteroidetes* (Rovella et al., 2021). Compared to healthy controls, the gut microbiota of hypertensive patients showed the reduced abundance of *Lactobacilli* and increased the abundance of *Prevotella* and *Klebsiella species* compared with healthy controls (Al Samarraie et al., 2023). In addition, the increased abundance of *Prevotellaceae* and *Peptococcaceae* showed a correlation with stroke severity (Sorboni et al., 2022).

The inhibition of salidroside on disease-related microbiota growth shows its potential to help restore a balanced gut microbiota (Xie et al., 2020). In a research experiment utilizing a mouse model with gut microbiota disturbance induced by antibiotics, the effects of salidroside on promoting the restoration of gut microbiota richness, diversity, and community structure were examined (Sun et al., 2022). Specifically, it was noted that salidroside stimulated the proliferation of advantageous genera such as *Bacteroides*, *Actinobacteria*, *Parabacteroides*, *Lactobacillus*, and *Bifidobacterium*. This ability to promote the growth of beneficial bacteria, particularly *Lactobacillus* and *Bifidobacterium*, suggests that salidroside may have prebiotic functions that can offer various health benefits to the host (Sun et al., 2022). Additionally, salidroside has been shown to inhibit the growth of disease-associated genera including *norank_f_Muribaculaceae*, *Helicobacter*, and *Ruminococcus_torques_group*. By balancing the microbiota composition, salidroside has the potential to contribute to the maintenance of a healthy gut microbiota. Treatment with salidroside supplement for 15 days resulted in a changed gut microbiota composition, along with the regeneration of the liver in mice exposed to furan (Yuan et al., 2019). It was notable that 20 mg/kg/day salidroside administration remarkably enhanced the abundance of phyla *Verrucomicrobia* suppressed by LPS and reduced the abundance of phyla *Proteobacteria* promoted by LPS. Experiments administering salidroside (100 mg/kg/day) to mice for 4 weeks revealed that salidroside increased the relative abundance of beneficial microbes: *Alistipes, Rikenellaceae, Parabacteroides,* and *Lactobacillus*, while decreasing the relative abundance of harmful bacteria such as *Candidatus Arthromitus* (Zhu et al., 2023). Wang *et al.* sought to determine whether salidroside could mitigate the symptoms of colitis induced by dextran sulfate sodium (DSS)-induced colitis (Wang et al., 2021). Mice with colitis were orally administered salidroside at doses of 125, 250, and 500 mg/kg/day for 14 days. Salidroside treatment could reverse the microbial dysbiosis induced by DSS, leading to an increase in abundance of *Turicibacter*, *Lactobacillus*, *Clostridia_UCG-014*, *Bifidobacterium*, *Bacteroides_acidifaciens*, and *Bacteroides_thetaiotaomicron* in the salidroside-treated groups. Conversely, levels of *Staphylococcus*, *Prevotellaceae_UCG-001*, *Parasutterella*, and *norank_f_norank_o_Clostridia_UCG-014* were decreased.

Disruption of the gut microbiota exacerbates inflammation, which is crucial in starting and advancing atherosclerosis (Sun et al., 2021). Although it is currently unclear how salidroside exerts its protective effects against atherosclerosis through modulation of gut microbiota-mediated inflammation, several studies have been conducted in other models of chronic inflammatory diseases. Inflammatory bowel disease (IBD), characterized as a state of chronic inflammation, confers a higher risk of developing ASCVD (Stone, 2020). A lot of studies discovered that IBD could be treated by salidroside through regulating the gut microbiota. Systemic inflammation in patients with IBD contributes to oxidative stress and increased levels of inflammatory cytokines, like TNF-α, that can lead to the development of atherosclerosis and cardiovascular disease (Jucan et al., 2022).

**FIGURE 2:**
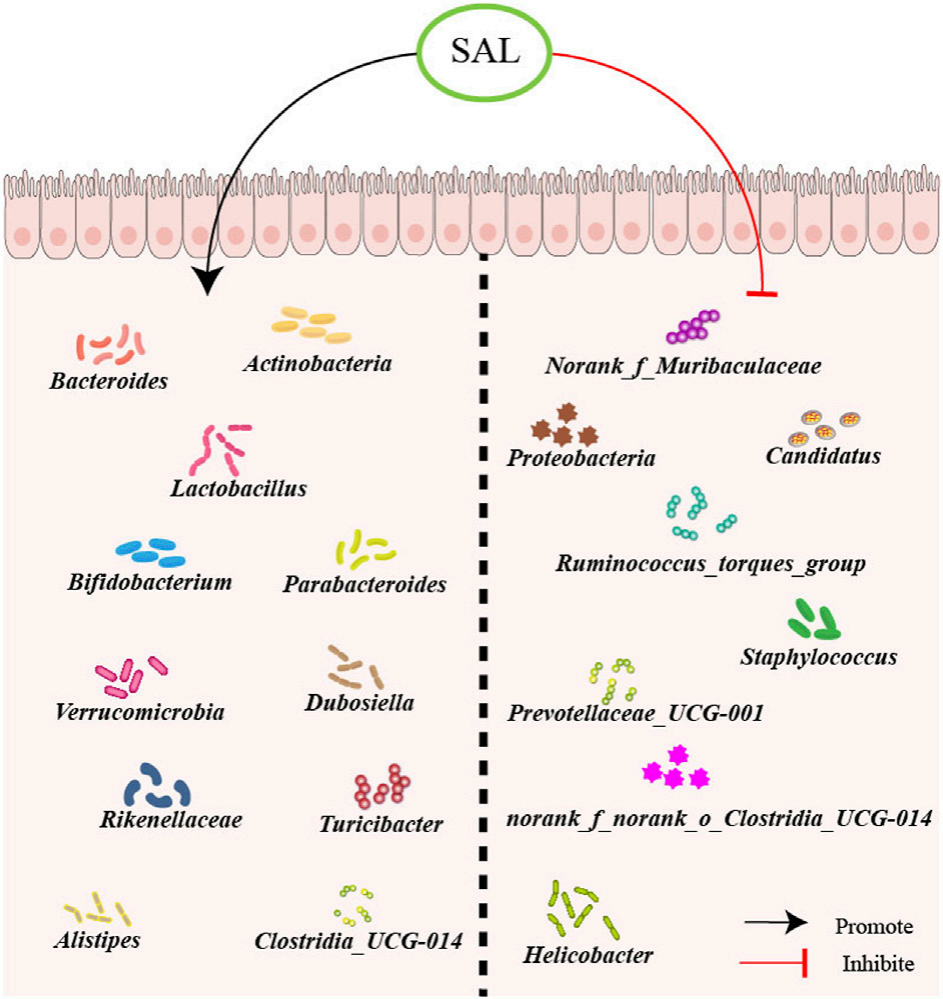
Schematic overview of salidroside on gut microbiota. First, salidroside could increase the abundance of beneficial bacteria, including *Norank_f_Muribaculaceae*, *Proteobacteria*, *Candidatus*, *Ruminococcus_torques_group*, *Staphylococcus*, *Prevotellaceae_UCG-001*, *norank_f_norank_o_Clostridia_UCG-014*, and *Helicobacter*. Besides, salidroside could reduce the abundance of harmful bacteria, like *Actinobacteria*, *Bacteroides*, *Lactobacillus*, *Bifidobacterium*, *Parabacteroides*, *Verrucomicrobia*, *Dubosiella*, *Rikenellaceae*, *Turicibacter*, *Alistipes* and *Clostridia_UCG-014*.

Salidroside as a small polyphenolic molecule can interfere with or degrade RNA (Birtić and Kranner, 2006), which may interfere with the result of gut microbiota. However, nowadays most studies on intestinal flora use the 16S rDNA method for detection, and the experimental results have a certain degree of reliability. The potential for salidroside to mitigate the progression of atherosclerosis through the manipulation of gut microbiota is a promising prospect. However, it is essential to conduct additional research, including human clinical trials, to substantiate the efficacy and understand the therapeutic mechanisms of salidroside in regulating gut microbiota (Figure 2).

## 5 Salidroside and gut microbiota-dependent metabolites

Accumulating evidence suggests that metabolites produced by the gut microbiome, such as trimethylamine N-oxide (TMAO), lipopolysaccharide (LPS), and short-chain fatty acids (SCFAs), impact cardiovascular health (Kasahara and Rey, 2019; Anto and Blesso, 2022; Cao et al., 2023). TMAO can promote the progression of atherosclerosis through cholesterol accumulation, pro-inflammatory pathways activation, endothelial dysfunction and thrombosis (Zhen et al., 2023). LPS enhances atherosclerosis by stimulating chronic systemic inflammation (Wang et al., 2022). Meanwhile, SCFAs show anti-atherosclerosis effects, including gut immune system modulation, intestinal barrier restoration, vascular inflammation inhibition, and oxidative stress amelioration (Yao et al., 2022). Salidroside alleviates LPS-induced human umbilical vein endothelial cells (HUVECs) injury by activation of autophagy and inhibition of the NLRP3 pathway (You et al., 2021). Salidroside could increase SCFAs production, which could contribute to the repair of antibiotic-induced intestinal damage (Sun et al., 2022). According to the above, salidroside may play the role of arteriosclerosis by regulating the metabolites of the intestinal microbiome, such as TMAO, LPS, etc.

### 5.1 Trimethylamine N-oxide

TMAO is a crucial metabolite produced by gut microbes (Thomas and Fernandez, 2021). The main classical pathways of TMAO are as follows (Zhu et al., 2020; Shi et al., 2022): Trimethylamine (TMA), the precursor of TMAO, is mainly absorbed through dietary phosphatidylcholine, betaine, choline and L-carnitine produced by some intestinal bacteria into the hepatic hilar circulating blood. TMA is efficiently absorbed and quickly metabolized by the liver enzyme flavin monooxygenase 3 (FMO3), resulting in the synthesis of TMAO. Recent research indicates that TMAO plays a crucial role in the progression of atherosclerotic events like atherosclerosis, myocardial infarction, thrombosis, arrhythmias, and stroke (Hardin et al., 2019; Wu et al., 2020; Manolis et al., 2022; Cao et al., 2023).

Phosphatidylcholine is the common dietary source of choline and trimethylamine oxide (Wang et al., 2011). A recent study suggested that phosphatidylcholine could be the lipid target in atherogenic mice that are controlled by salidroside (Wen et al., 2020). They analyzed that the lipid species from hepatic extracts in ApoE^−/−^ mice and discovered the level of phosphatidylcholine could be ameliorated by salidroside. On the other hand, the concentration of TMAO was associated with increased activity of the *Firmicutes* and *Proteobacteria* which were considered as the producer of TMAO (Arias et al., 2020). In a study by Jing et al., they found that salidroside (1.5 g/kg, 5 weeks) could decrease the level of *Firmicutes* and *Proteobacteria* in db/db mice (Shi et al., 2022). A separate investigation reached a similar conclusion that salidroside decreased the levels of *Firmicutes* and *Proteobacteria* in the gut microbiome of mice with colitis (Wang et al., 2021). In short, TMAO production is associated with phosphatidylcholine levels and the activity of *Firmicutes* and *Proteobacteria*. Salidroside shows promise in potentially reducing TMAO production by targeting these factors. Therefore, we propose that salidroside may have a beneficial effect on atherosclerosis by decreasing TMAO levels. However, further *in vivo* and *in vitro* studies are necessary to confirm this hypothesis.

### 5.2 Lipopolysaccharides

LPS is a potential contributor to atherosclerosis (Suzuki et al., 2022). Yoshida et al. detected that the LPS levels in patients with CAD were negatively correlated with the abundance of *Bacteroides vulgatus* and *Bacteroides dorei* (Yoshida et al., 2018). By transporting pro-atherogenic lipoproteins, including very low-density lipoprotein (VLDL) and low-density lipoprotein (LDL), LPS plays a critical role in the initiation and progression of atherosclerosis (Gorabi et al., 2022). Additionally, it can enhance the synthesis of pro-inflammatory cytokines like as interleukin (IL)-8 and tumor necrosis factor (TNF), leading to acute inflammation and the subsequent creation of neutrophil extracellular traps (NETs) that may destabilize atherosclerotic plaques (Violi et al., 2023a). Abnormalities in the gut microbiota led to LPS secretion, disruption of gastrointestinal permeability (Kesika et al., 2021), and gut bacterial translocation (Li et al., 2020). The movement of LPS into the bloodstream is caused by heightened intestinal permeability. It can promote chronic inflammation and represents a major risk factor for the development of atherosclerosis. Therefore, LPS plays a pivotal role in the interaction between intestinal microbiota and ASCVD.

Treatment with salidroside at doses of 10, 20, and 40 mg/kg/day was found to be more effective in suppressing LPS levels and inhibiting systemic low-grade inflammation compared to the furan-treated group. They found that salidroside could upregulate LPS-suppressing genera *Akkermansia* and downregulate LPS-producing phyla Proteobacteria (Yuan et al., 2019). Besides, salidroside could increase the abundance of *Bacteroides*, which might decrease the level of LPS (Xie et al., 2020). The increase in *Bacteroidetes* abundance leading to lower LPS levels may be attributed to the immune regulatory capabilities of *Bacteroides*, which exhibit anti-inflammatory properties and help preserve the integrity of the intestinal epithelium (Tan et al., 2019).

What’s more, some studies demonstrated that salidroside reduced the production of TNF-α and IL-6 induced by LPS (Song D. et al., 2021; Jiang et al., 2022). Chen et al. (2017) discovered that salidroside (20 and 40 mg/kg/d, 3 days) might ameliorate LPS-induced inflammatory cytokines through the inhibition of ROS-mediated PI3K/AKT/mTOR pathway. Another study found that salidroside could reduce the levels of IL-1β, IL-6, TNF-α, and IL-18 in cells induced by LPS. The study (Tan et al., 2022) also demonstrated that salidroside effectively reversed the inflammatory response by increasing the expression of tumor necrosis factor-inducible protein 8-like protein 2 (TNFAIP8L2) and reducing the expression of the high-mobility group box1 (HMGB1)/Toll-like receptor 4 (TLR4)/nuclear factor-κB (NF-κB) signaling pathway. Furthermore, salidroside was shown to inhibit the activation of proinflammatory macrophages and the production of cytokines like monocyte chemoattractant protein 1 (MCP1), TNF-α, IL-1β, and IL-6 in LPS-ethanol-induced cells (Li et al., 2019). Testing salidroside at different concentrations (25, 50, and 100 μg/mL) on human monocyte-like cells (THP-1) stimulated with LPS and ethanol revealed its inhibitory effects on pro-inflammatory mediators through the Notch signaling pathway blockade.

**FIGURE 3:**
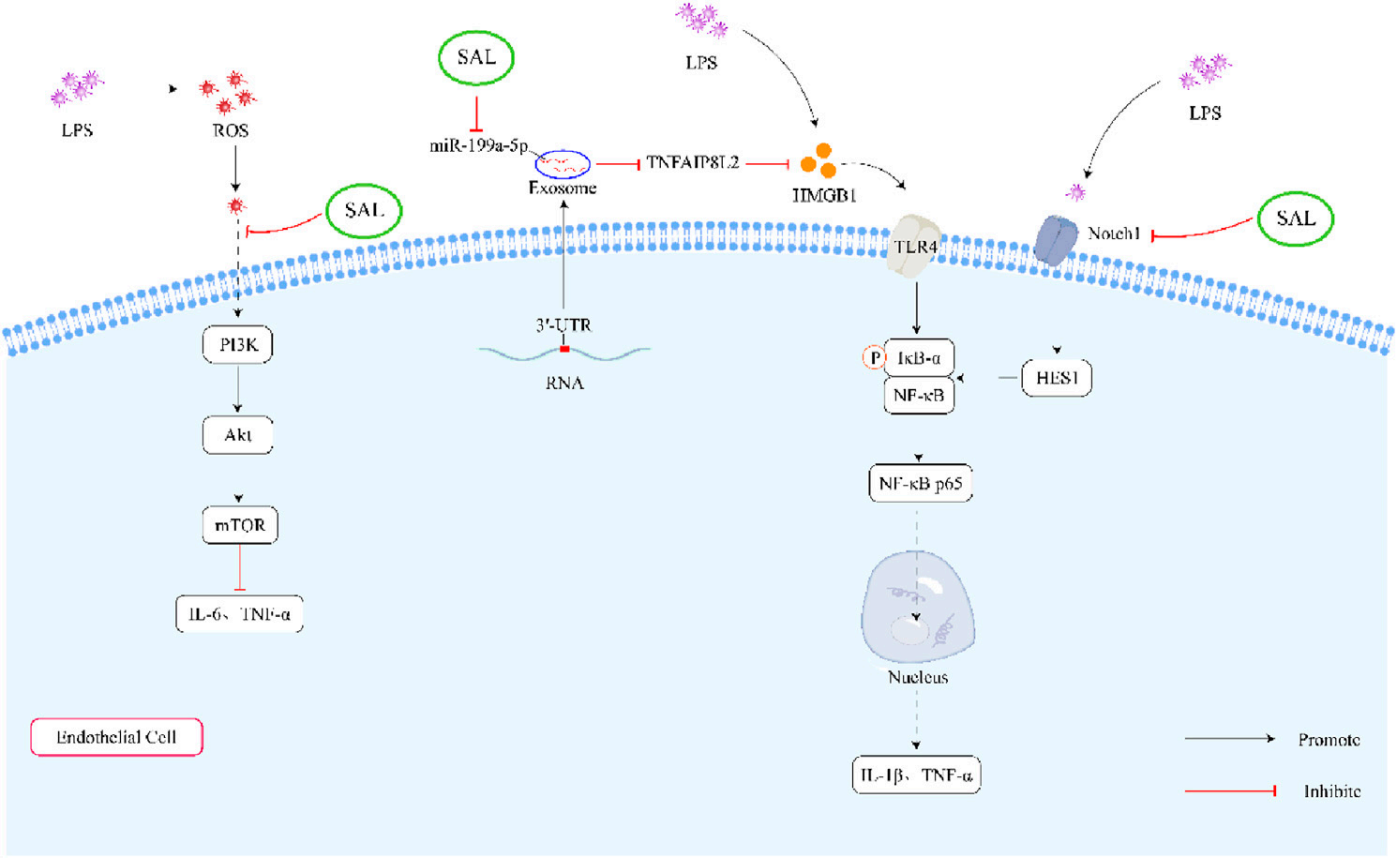
Schematic representation of the effects of salidroside on LPS-induced inflammation. First, salidroside can alleviate the production of IL-6 and TNF-α through inhibiting ROS-mediated PI3K/AKT/mTOR signaling pathways. Secondly, salidroside can decrease the concentration of IL-1β and TNF-α by reducing the overexpression of exosome miR-199a-5p. What’s more, salidroside might decrease the LPS-induced inflammatory cytokines through inhibiting the Notch-Hes signaling pathway.

All in all, salidroside could inhibit the LPS-induced inflammatory cytokines through the PI3K/AKT/mTOR pathway, HMGB1/TLR4/NF-κB signaling pathway and Notch signaling pathway (Figure 3).

### 5.3 Short-chain fatty acids

SCFAs (the number of carbon chains < 6) are mainly produced during the bacterial fermentation of dietary fiber in the intestinal tract (Liu et al., 2021). Acetate, propionate, and butyrate are the primary components of SCFAs, which play a crucial role in shielding the gut epithelium and inhibiting bacterial translocation into the bloodstream (Shen et al., 2021). A study conducted on the metabolic profile and plasma fatty acid network in patients with AMI found that the proportion of SCFA was notably reduced in 290 AMI patients compared to individuals in good health (Guo et al., 2021). This result suggested that SCFA% exhibited a potential diagnostic value in AMI. A prospective study was carried out with Chinese acute ischemic stroke patients, focusing on the correlation between gut microbiota and fecal SCFAs (Haghikia et al., 2022). The research validated that there was a decrease in SCFAs levels in the intestines of acute ischemic stroke patients, which correlated with a higher likelihood of experiencing unfavorable functional outcomes after 90 days (Tan et al., 2021). Accumulating evidence has suggested that SCFAs have the beneficial effects of decreasing the risk of CAD (Chen et al., 2020; Hu et al., 2022). The study aimed to evaluate the effects of propionate supplementation on hypercholesterolaemic subjects (Haghikia et al., 2022). A total of 62 individuals with LDL cholesterol levels above 115 mg/dL were recruited for the study. The participants were randomized into two groups: one group received a placebo while the other group received 500 mg of propionate twice daily for 8 weeks. They found that propionate lowered LDL cholesterol levels and total cholesterol levels compared to placebo. What’s more, they found the same conclusion in ApoE^−/−^ mice (Bartolomaeus et al., 2019). In addition, Ma et al. established that butyrate could suppress chronic atherosclerotic inflammation in ApoE^−/−^ mice (Ma et al., 2023). Butyrate reduces ROS and various inflammatory markers, lower overall cholesterol levels, and controls the growth and movement of smooth muscle cells (Xiao et al., 2021). This helps to slow down the progression of atherosclerosis.

Salidroside treatment for 7 days reversed the attenuation of SCFAs in antibiotic-treated mice, leading to a significant increase in SCFAs contents (Sun et al., 2022). The researchers hypothesized that salidroside might enhance the abundance of butyric acid-producing bacteria, such as *Odoribacter*, *Anaerotruncus*, *norank_f_Ruminococcaceae*, *unclassified_f_Lachnospiraceae*, *norank_f_Lachnospiraceae*, and *Eubacterium_fissicatena_group*. From improving barrier functions to reducing inflammation and aiding in the repair of damage, SCFAs play a crucial role in maintaining a healthy gut environment. This increase of SCFAs potentially improves intestinal barrier functions, helps combat inflammatory responses, and aids in the repair of intestinal damage. In a study conducted by Song et al. (2021b), it was shown that salidroside treatment (25 mg/kg/day) led to a significant upregulation of 3-hydroxybutyrate levels in ApoE^−/−^ mice. Butyric acid is essential for ensuring the proper function of the intestinal barrier, strengthening the defense barrier, and regulating immune responses and inflammation (Liu et al., 2021). These results show the potential advantages of salidroside in supporting gut health and overall wellness by influencing SCFAs, specifically butyric acid.

Salidroside has the potential to increase SCFAs levels in the body, which may help to mitigate the progression of atherosclerosis and improve overall cardiovascular health. However, further research is required to fully understand how salidroside impacts the expression of SCFAs.

## 6 Other complex interactions

### 6.1 Integrity of the epithelial barrier function

Evidence for the contribution of intestinal barrier injury to the development of atherosclerosis is accumulating. With the increasing intestinal permeability, the circulating level of LPS is increasing (Wang et al., 2022). Increased intestinal barrier permeability leads to low-grade endotoxemia, which damages the arterial wall and promotes the progression of atherosclerosis (Violi et al., 2023b). The studies that have shown that salidroside may attenuate intestinal barrier injury are as follows.

The intestinal barrier is a crucial interface that separates the external environment from the internal environment of the body. The intestinal epithelium serves a dual function of facilitating nutrient absorption and protecting against harmful substances (Schoultz and Keita Å, 2020). The epithelial cytoskeleton, composed of scaffold proteins like ZO-1 and transmembrane proteins such as occludin, junctional adhesion molecules, and claudins, plays a crucial role in maintaining the integrity of the gut mucosal barrier function (Alizadeh et al., 2022). *Rhodiola crenulata* extract (Sanchez-Rodriguez et al.), of which salidroside is one of the main components, significantly upregulated ZO-1 and occludin expressions in the colon of colitic mice (Wang et al., 2021). As the decrease of ZO-1 and occludin expressions and the elevated LPS could be reversed by salidroside, it indicates that intestinal barrier function is partially repaired by salidroside (Liu et al., 2019; Wang et al., 2021).

Salidroside has been found to have the ability to restore the decreased expression of ZO-1, occludin and claudin-5. This reversal of protein expression helps to prevent intestinal damage and protect the intestinal mucosal barrier during sepsis. The mechanism by which Salidroside achieves this protective effect is through regulating IL-17 levels, which in turn blocks the NF-κB and p38 MAPK signaling pathways (Liao et al., 2023). In LPS-activated intestinal epithelial cells, salidroside could suppress the secretion of pro-inflammatory cytokine IL-6 through attenuating the NF-κB, MAPK, and JAK-STAT3 signaling pathways (Wang et al., 2019). What’s more, they discovered that salidroside could maintain the expressions of human defensins (HD)-5 and HD-6 in intestinal epithelial cells, which could protect the mucosal intestinal barrier function (Ehmann et al., 2019). Compared with the furan group, salidroside could increase the expression of Occludin and ZO-1 in the colon (Yuan et al., 2020). In addition, salidroside could improve the production of butyrate (Song et al., 2021), which is confirmed as an available factor in improving gut barrier function and intestinal permeability (Chen et al., 2020).

**FIGURE 4:**
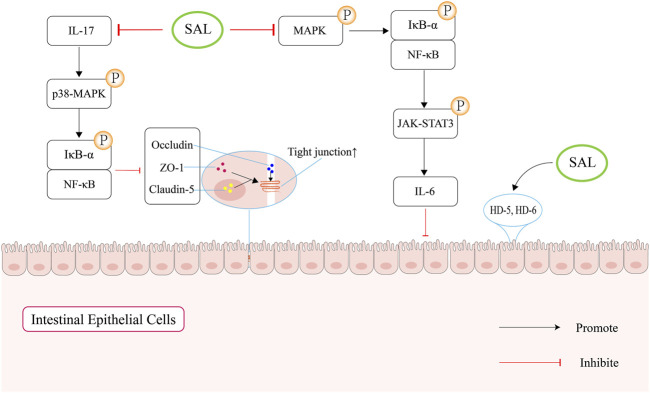
Schematic overview of salidroside-mediated enhancement of the intestinal mucosal barrier function. Firstly, salidroside can increase the levels of intestinal tight junction proteins by regulating IL-17 to block the NF-κB and p38 MAPK signaling pathways. Second, salidroside can attenuate the NF-κB/MAPK/JAK-STAT3 signaling pathways, which can suppress the secretion of IL-6. In addition, salidroside can increase the expression of human defensin (HD)-5 and HD-6, which can protect the mucosal intestinal barrier function.

In summary, salidroside can maintain gut mucosal barrier function through upregulating the expression of ZO-1, occludin and butyrate, and suppressing the secretion of pro-inflammatory cytokine (Figure 4).

### 6.2 NLRP3 and inflammation

Atherosclerosis is a chronic inflammatory disease of the vascular walls. The inflammatory signaling pathways involved in the occurrence and progression of atherosclerosis, including the NLRP3 inflammasome receptor, Toll-like receptor, proprotein convertase subtilisin/kexin type 9 (PCSK9), Notch and Wnt signaling pathways (Kong et al., 2022). In particular, the levels of NLRP3 inflammasome components such as Caspase-1, IL-1β, and IL-18 are increased in coronary atherosclerotic lesions, suggesting that NLRP3 plays an important role in the occurrence and development of atherosclerosis (Sharma and Kanneganti, 2021). In addition, increased NLRP3 expression is positively associated with the severity of coronary artery stenosis (Silvis et al., 2021).

Research demonstrates the crucial role of NLRP3, a multi-protein complex located inside cells, in the inflammatory response and recruitment of inflammatory cells in the body. It is crucial for maintaining the body’s immune function and the balance of the intestinal microbiome (Yao et al., 2017). The activation and assembly of NLRP3 inflammasomes are intricately linked to the gut microbiota composition and intestinal metabolites, which may lead to alterations in intestinal dysbiosis and other functionalities (Wen et al., 2020; Song et al., 2021; Li et al., 2021). The gut microbiome and its metabolites modulate the NLRP3 inflammasome activation, which can worsen intestinal dysbiosis by altering the *Firmicutes*/*Bacteroidetes* ratio and increasing *Prevotellaceae* levels (Pellegrini et al., 2020). NLRP3 can be activated by TMAO, which can induce vascular inflammation and drive endothelial dysfunction (Zhang et al., 2022).

A study discovered that mice lacking NLRP3 demonstrated less inflammation, lower bile acids, and modified fatty acid (FA) expression (Aparicio-Ugarriza et al., 2020). These alterations coincided with shifts in the gut microbiota composition, which correlated with decreased systemic levels of TMAO and LPS. Consequently, this could potentially lower systemic inflammation and positively impact lipid metabolism (Chang et al., 2007). Studying the relationship between the NLRP3 inflammasome and gut microbiota in atherosclerosis is vital for progress in preventing and treating this disease.

Salidroside has been shown to possess anti-inflammatory and antioxidant properties by targeting the TLR4/MyD88/NF-κB-dependent pyroptosis pathway, both *in vivo* and *in vitro* (Zhang et al., 2020). A separate investigation concluded that salidroside (2, 10, 50 μM) could reverse the increased levels of cleaved Caspase-1, IL-1β, and IL-18, which are downstream targets of NLRP3, leading to inhibition of pyroptosis (Cai et al., 2021). Moreover, research by Hu et al. demonstrated that salidroside could alleviate endothelial inflammation and oxidative stress by modulating the AMPK/NF-κB/NLRP3 signaling axis (Hu et al., 2020). They treated HUVECs with salidroside at 10, 50, or 100 μM for 24 h. They found that salidroside could decrease the level of proinflammatory factors, such as IL-1β, IL-6, and TNF-α, as well as reduce ROS production. It has been established that salidroside has the ability to prevent pyroptosis and inhibit NLRP3-mediated pyroptosis through the suppression of the P2X7/NF-κB/NLRP3 signaling pathway (Chai et al., 2022).

**FIGURE 5:**
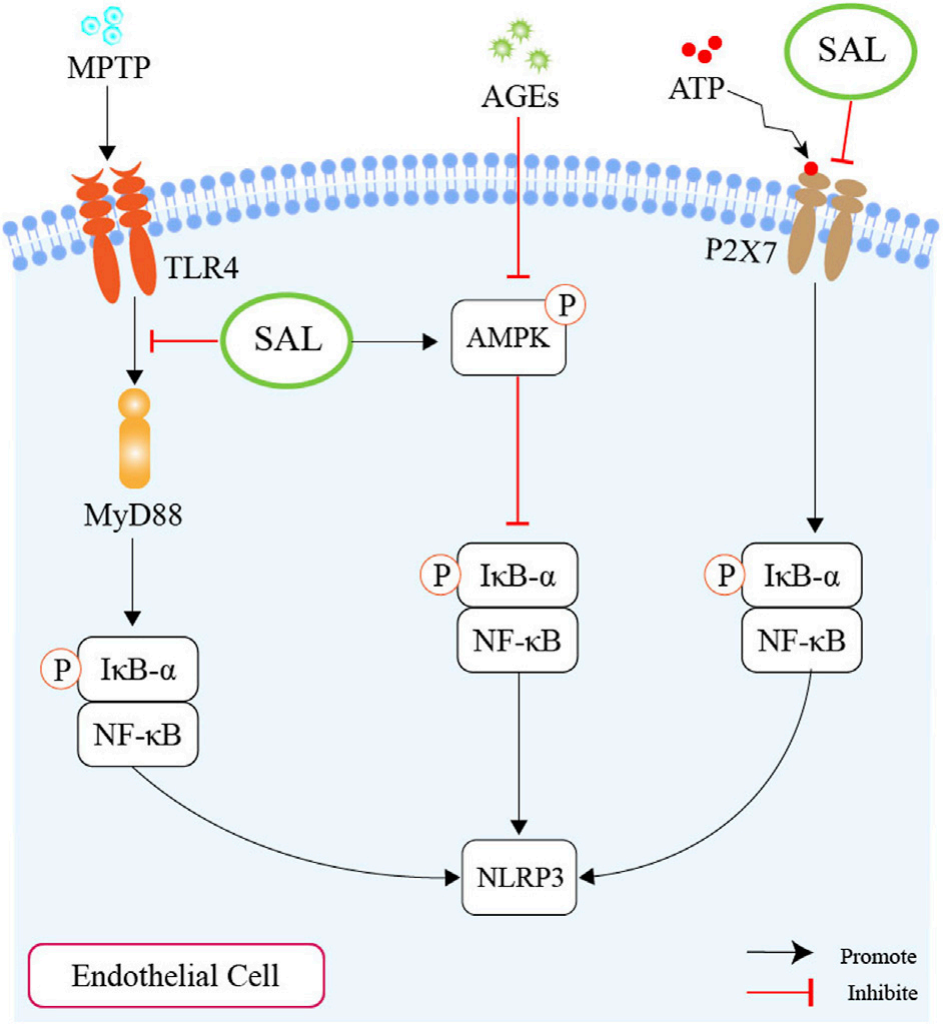
Schematic overview of salidroside on inhibiting the production of NLRP3. First of all, salidroside could inhibit the level of NLRP3 through the TLR4/MyD88/NF-κB-dependent pyroptosis pathway. Second, salidroside could alleviate endothelial inflammation and oxidative stress by modulating the AMPK/NF-κB/NLRP3 signaling axis. Thirdly, salidroside could inhibit pyroptosis through the suppression of the P2X7/NF-κB/NLRP3 signaling pathway.

As mentioned above, we plausibly speculate that salidroside might ameliorate atherosclerosis by inhibiting the NLRP3-associated gut-coronary axis (Figure 5).

## 7 Conclusion and future perspectives

Recent evidence has been a growing body of evidence highlighting the significance of gut microbiota in the development and advancement of atherosclerosis (Kazemian et al., 2020; Li et al., 2024b; Mao et al., 2024). *Oscillatory* spp. has been shown to efficiently absorb cholesterol from various intestinal sources in humans and convert this cholesterol into ketones, glycosylated cholesterol, and hydroxycholesterol, which may impact lipid homeostasis and cardiovascular health (Li et al., 2024). Mendelian randomization studies in two samples suggested that complex networks may exist among gut microbes, and interactions between bacteria, viruses, and fungi jointly influence the occurrence and progression of atherosclerosis (Jiang et al., 2024). *Desulfovibrioceae* showed a stable and significant negative correlation with ApoB levels (Teng et al., 2024), which was associated with increased TG levels (Takeda et al., 2023). The traditional Chinese herbal prescription (Yu et al., 2024), serum sex hormones (Peters et al., 2023), and structure of the gut virome (Li et al., 2024c) play an important role in slowing down the progression of atherosclerosis by regulating altered intestinal microbiota and perturbed metabolites.

The mechanisms by which salidroside could regulate the intestinal microbiome in atherosclerosis are as follows: 1) Salidroside has been shown to have a positive impact on the gut microbiome by promoting the growth of beneficial bacteria and suppressing the growth of harmful bacteria. 2) Salidroside can reduce liver phosphatidylcholine absorption, which can suppress the production of TMAO by gut bacteria. 3) By obstructing the ROS-mediated PI3K/AKT/mTOR pathway and impeding the Notch signaling pathway, salidroside can inhibit the production of LPS. 4) Salidroside has the capability to elevate the production of SCFAs, specifically butyric acid and acetic acid. 5) By elevating the levels of ZO-1 and occludin proteins, salidroside could enhance the integrity of the intestinal barrier and enhance intestinal permeability. 6) Salidroside may reduce the activation of NLRP3 to protect the balance of intestinal microecological, further reducing systemic levels of TMAO and LPS.

Salidroside has the ability to directly reshape the gut microbiota (Shi et al., 2022) and also indirectly influence them through reducing inflammation (Liu et al., 2023), enhancing gut and mucus layer integrity (Wang et al., 2021), promoting appropriate immune responses (Yang et al., 2024), and increasing antimicrobial peptide production (Soroudi et al., 2024). However, there is currently a lack of *in vitro* studies investigating whether and how salidroside selectively alters bacterial function or competitiveness. *In-vitro* models of the human gut microbiota, such as Transwell culture models (Biagini et al., 2023), could be an effective approach to delineate the specific mechanisms through which salidroside protects against atherosclerosis by interacting with the gut microbiota and its metabolites.

In conclusion, the anti-atherosclerotic effect of salidroside can be partially attributed to its influence on intestinal microbiota and metabolites, which leads to a decrease in systemic inflammation and modulation of lipid metabolism. While research has shown that salidroside can alter the gut microbiota in animal models, a definitive connection between salidroside’s effects on gut microbiota and atherosclerosis remains unclear. Furthermore, further research is necessary to delve into the specific pathways through which salidroside protects against atherosclerosis by interacting with the gut microbiota and its metabolites. These studies will help enhance our understanding of the mechanisms underlying the beneficial effects of salidroside in preventing atherosclerosis (Table 1).

**TABLE 1:** The anti-atherosclerotic targets of salidroside.

Targets	Potential pathway	Effect
Gut microbiota	Beneficial bacteria	Upregulate
	Harmful bacteria	Downregulate
TMAO	*Firmicutes* and *Proteobacteria*	Downregulate
LPS	PI3K/AKT/mTOR pathway	Downregulate
	HMGB1/TLR4/NF-κB signaling pathway	Downregulate
	Notch signaling pathway	Downregulate
SCFAs	Butyric acid-producing bacteria	Upregulate
Integrity of the epithelial barrier function	NF-κB, MAPK, and JAK-STAT3 signaling pathways	Downregulate
	(HD)-5 and HD-6	Upregulate
	NF-κB and p38 MAPK signaling pathways	Downregulate
NLRP3	TLR4/MyD88/NF-κB-dependent pyroptosis pathway	Downregulate
	AMPK/NF-κB/NLRP3 signaling pathway	Upregulate
	P2X7/NF-κB/NLRP3 signaling pathway	Downregulate
